# Editorial: Brain cytoprotection for reperfusion injury after acute ischemic stroke

**DOI:** 10.3389/fneur.2026.1893024

**Published:** 2026-06-19

**Authors:** Lujia Tang, Zilong Zhao, Wenbo Zhao, Léon Ikka, Shuling Liu, Ming Wei

**Affiliations:** 1 Huanhu Hospital Affiliated to Tianjin Medical University, Tianjin Medical University, Tianjin, China; 2 Department of Neurosurgery, Tianjin Huanhu Hospital, Tianjin, China; 3 Tianjin Key Laboratory of Cerebral Vascular and Neurodegenerative Diseases, Tianjin Huanhu Hospital, Tianjin, China; 4 Tianjin Key Laboratory of Cerebral Blood Flow Reconstruction and Head and Neck Tumor New Technology Translation, Tianjin Huanhu Hospital, Tianjin, China; 5 Department of Neurosurgery, Tianjin Neurological Institute, State Key Laboratory of Experimental Hematology, Tianjin Medical University General Hospital, Tianjin, China; 6 Department of Neurology, Xuanwu Hospital, Capital Medical University, Beijing, China; 7 Beijing Key Laboratory of Hypoxic Conditioning Translational Medicine, Xuanwu Hospital, Capital Medical University, Beijing, China; 8 NEURI Brain Vascular Center, Bicêtre Hospital, Interventional Neuroradiology, Le Kremlin-Bicêtre, France

**Keywords:** acute ischemic stroke, cytoprotection, neurovascular protection, reperfusion after ischemia, reperfusion injury (IRI)

Acute ischemic stroke (AIS) remains a leading cause of death and long-term disability worldwide. Although the new era of reperfusion therapies such as thrombolysis and thrombectomy has significantly improved outcomes, many patients fail to achieve meaningful recovery due to reperfusion injury, a paradoxical process that involves a complex cascade of excitotoxicity, oxidative stress, blood–brain barrier disruption, mitochondrial dysfunction, apoptosis, and neuroinflammation, which together limit the benefits of recanalization. Thus, brain cytoprotection should not be viewed as an optional adjunct to reperfusion, but as a central strategy for extending the biological benefit of recanalization. This Research Topic, “*Brain Cytoprotection for Reperfusion Injury after Acute Ischemic Stroke*,” brings together clinical, translational, and mechanistic studies that collectively reframe reperfusion injury as a targetable continuum rather than a single downstream complication. By integrating these insights, this topic aims to support the development of effective strategies that enhance neurovascular protection and improve functional outcomes after stroke.

## Risk factors, stratification and early identification

A recurring message across this Research Topic is that cytoprotection begins before injury becomes clinically irreversible. Even after technically successful recanalization, unfavorable outcomes remain common and heterogeneous, ranging from a minority of patients with mild thrombolysis-treated stroke to more than half of selected thrombectomy cohorts. This variability underscores the need to move beyond a one-size-fits-all approach and toward earlier, more individualized risk stratification. The first study by Cao et al. integrated clinical parameters including admission, 24-h NIHSS scores, diastolic blood pressure, and coronary heart disease as key predictors of poor 90-day outcomes and developed a nomogram for individualized prognostic assessment after thrombolysis, illustrating how routinely available clinical variables can be translated into individualized prognostic tools. In addition to clinical indicators, metabolic and biochemical markers have emerged as important predictors of stroke prognosis. Gao et al. demonstrated that serum ferritin (SF), which reflects iron storage and inflammation, is closely associated with initial stroke neurological deficit, suggesting a potential link between metabolic dysregulation and early brain injury. The triglyceride-glucose (TyG) index, a surrogate marker for insulin resistance, was introduced by Sun et al. where they showed that TyG index was significantly associated with poor outcomes in AIS patients after thrombectomy (EVT) and may serve as a valuable prognostic marker for risk stratification. Similarly, Li et al. identified TyG index as an independent predictor of early neurological improvement, and its predictive performance was further enhanced when combined with baseline NIHSS scores. Together, these studies suggest that early prognostication should integrate neurological severity, vascular risk, metabolic stress rather than a single clinical scale.

## Reperfusion therapies and complications

The studies in this section remind us that reperfusion is not a purely mechanical endpoint. It is a biological intervention that may generate procedure-related and systemic undesirable consequences. The first study conducted by Xiang et al. investigated whether early prophylactic heparin use was associated with reduced short- and long-term mortality in patients with non-traumatic subarachnoid hemorrhage with a dose–response relationship. Xu et al. investigated the prognostic value of pre-procedural vitamin D levels in patients with acute anterior circulation large vessel occlusion who underwent successful MT. They found that vitamin D deficiency was independently associated with poor 3-month functional outcomes and increased mortality. Besides, some interventions targeting reperfusion-related complications are emerging. Gao et al. conducted a double-blind, randomized, placebo-controlled trial to evaluate the role of low-dose dexmedetomidine in the cerebral hyperperfusion syndrome (CHS) after AIS. They found that dexmedetomidine significantly reduced the incidence of CHS and postoperative pain scores following thrombectomy. Wang et al. described an *in situ* post-ischemic conditioning (IPC) strategy after successful thrombectomy in AIS. This modified technique was designed to reduce ischemia–reperfusion injury by applying repeated short cycles of balloon inflation and deflation at the original occlusion site. A common principle was highlighted that successful reperfusion requires not only vessel reopening, but also protection against the physiological instability that follows.

## Early neurological changes

Early neurological evolution is more than a short-term clinical observation. It is an early window into the success or failure of reperfusion biology. Patients with similar angiographic outcomes may diverge rapidly in neurological trajectory, indicating that recanalization, tissue salvage, inflammation, edema, and systemic vulnerability interact in complex ways. The first study conducted by Miao et al. revisited the application of Oxford Community Stroke Project (OCSP) classification, a widely used clinical tool for rapid stroke classification. The accuracy of its early decision-making can be improved by integration with neuroimaging and functional evaluation compared with its use alone. Furthermore, several studies focus on the early neurological evolution after reperfusion. A machine learning-based model that incorporates clinical and biochemical variables was established by Li et al. to predict early neurological improvement (END) in patients after mechanical thrombectomy (MT). Lv et al. developed a machine learning-based nomogram to predict early neurological improvement after intravenous thrombolysis which combined the clinical and biochemical markers to achieve the best predictive performance. In contrast, Zhao et al. demonstrated that admission NIHSS score and diabetes are key predictors of early neurological improvement (ENI), emphasizing the role of baseline neurological status and metabolic comorbidities in the early prognostication. These studies collectively argue that early neurological assessment should be treated not merely as an outcome measure, but as a dynamic phenotype that can guide risk monitoring and therapeutic escalation.

## Ischemia-reperfusion injury

Mechanistic studies in this topic reinforce the concept that ischemia-reperfusion injury is a neurovascular syndrome rather than a neuron-restricted event. Wang et al. showed that recanalization aggravates blood–brain barrier (BBB) permeability by impairing tight junction integrity and enhancing transcytosis, thereby promoting vascular leakage and increasing the risk of hemorrhagic transformation. In parallel, Olszewski et al. highlight that cerebral edema continues to evolve in the subacute phase after stroke and is not associated with prior administration of alteplase before EVT, but rather with the degree of reperfusion. These observations indicate that successful reperfusion should no longer be defined solely by restoration of vessel patency, but also by preservation of downstream microvascular integrity and neurovascular resilience. Neuroinflammation emerges as another key driver of reperfusion injury. Ouyang et al. demonstrated that inhibiting inflammatory pathways, such as NLRP3 inflammasome–associated pyroptosis and microglial inflammatory polarization, can reduce neuronal injury and improve neurological function. In addition, Song et al. found that M2 macrophage-derived exosomes were shown to suppress microglial activation and attenuate inflammatory responses, suggesting a potential therapeutic strategy based on exosomes. In line with this, Ma et al. analyzed the ferroptosis associated genes and showed the potential of exosomal circRNAs as diagnostic biomarkers for ferroptosis in cerebral ischemia–reperfusion injury (CI/RI), while excessive formation of neutrophil extracellular traps (NETs) can exacerbate vascular dysfunction and BBB damage. Chang et al. suggested that acupuncture targeting the tryptophan–indole–NETs axis may improve vascular integrity and enhance the efficacy of rt-PA thrombolysis. Taken together, these studies broaden the cytoprotection framework from direct neuronal rescue to integrated control of endothelial dysfunction, immune activation, ferroptosis, extracellular vesicle signaling, and neutrophil-driven vascular injury.

## Integrated care, biomarkers and systems perspective

Several studies in this topic highlight the integrated approach encompassing structured care management and comprehensive perspective. Yang et al. reviewed the early identification and integrated nursing management of hemorrhagic transformation after intravenous thrombolysis in AIS. They proposed a multimodal early detection strategy, together with systematic nursing interventions to emphasize the importance of timely risk assessment and targeted interventions. In addition, Zhan and Xu evaluated the impact of structured emergency nursing interventions on pre-hospital and in-hospital outcomes in acute ischemic stroke. In this retrospective cohort study, system-level improvements in emergency stroke care significantly enhanced treatment efficiency. As systemic inflammation has emerged as a key determinant of stroke prognosis, Huang et al. found that composite hematological indices may serve as early risk stratification and individualized outcome prediction after thrombolysis. Lastly, in a systematic perspective, bibliometric analyses performed by Peng et al. further reveal acute brain injuries' research is increasingly evolving toward interdisciplinary collaboration, biomarker discovery, precision medicine, and global neurocritical care integration. These contributions indicate that future progress will depend not only on discovering new targets, but also on embedding them into timely, coordinated, and scalable clinical pathways.

Collectively, these studies demonstrate a central perspective: improving outcomes after AIS requires a transition from reperfusion-centered treatment to more cytoprotection-oriented care after recanalization. Recanalization remains indispensable, but its clinical value is ultimately determined by whether the downstream brain, vasculature, and immune environment can be preserved. Future progress will depend on integrating early risk stratification, mechanistic understanding of reperfusion injury, targeted neuroprotection, and comprehensive multidisciplinary care. Together, this Research Topic provides a roadmap for more precise and biologically informed stroke management.

